# The potential influence of human parainfluenza viruses detected during hospitalization among critically ill patients in Kuwait, 2013–2015

**DOI:** 10.1186/s12985-017-0681-0

**Published:** 2017-02-03

**Authors:** Sahar Essa, Haya Al-tawalah, Sarah AlShamali, Widad Al-Nakib

**Affiliations:** 1 Department of Microbiology, Faculty of Medicine, Kuwait University, Kuwait City, Kuwait; 2 Ministry of Health, Sabah Hospital, Virology Unit, Kuwait City, Kuwait; 3 Ministry of Health, Sabah Hospital, Kuwait City, Kuwait

**Keywords:** Parainfluenza viruses, Intensive Care Unit (ICU), Pediatric intensive care unit (PICU)

## Abstract

**Background:**

The four types of human parainfluenza viruses (PIV) are important causes of community-acquired pneumonia, particularly in children; however, limited information exists about the incidence of PIV in critically ill patients. The aim of this study is to describe the spectrum, incidence and clinical features of PIV-associated infections diagnosed during the hospital stay of patients admitted to pediatric intensive care unit (PICU) and intensive care unit (ICU) of 5 medical centers across Kuwait.

**Methods:**

This was a population-based, retrospective study from 2013 to 2015. Specimens were analyzed by molecular methods. This analysis was performed using the database of Virology Unit, Mubarak Al-Kabeer Hospital. Data from 1510 admitted patients with suspected respiratory viral infections was extracted.

**Results:**

The database contained a total of 39 (2.6%) patients infected with PIV (53.8% male and 46.2% females) and 20 (51.3%) were under 1 year of age. The most frequently isolated type was type 3 (28, 71.8%) followed by type 1 (9, 23.1%). At admission the most common clinical diagnosis was pneumonia in 12 patients (30.8%, *p* < 0.05) followed by bronchiolitis in 10 patients (25.6%).

**Conclusion:**

PIV plays an important yet unrecognized role in the outcomes of PIUC and ICU patients. Our results contribute to the limited epidemiologic data of PIV in PIUC and ICU in this region.

## Background

Viral infections are ubiquitous and are common in patients admitted to intensive care unit (ICU) and pediatric intensive care unit (PICU) and may be associated with significant morbidity and mortality [1]. The vast majority of scientific articles dealing with infections address bacterial or fungal infections, and viral agents are often disregarded. Despite their prevalence, viral infections are frequently not considered to be of clinical significance among the critically ill patients, unless the patient is immunocompromised. Among immunocompetent critically ill patients, viral infections can lead to a significant morbidity and mortality [2].

Parainfluenza viruses (PIV) were first discovered in 1950. They are enveloped non-segmented, negative single-stranded RNA viruses. PIV are genetically and antigenically divided into four types: PIV-1, PIV-2, PIV-3 and PIV-4, each with different genetic and antigenic features [3]. PIV are a cause of community-acquired pneumonia in healthy individuals and can infect individuals of any age group [4–6]. The majority of PIV-infected patients are treated in outpatient clinics, yet PIV infections are one of the most common causes of respiratory diseases leading to hospitalization [4, 5, 7]. Among the immunocompromised patients, PIV especially type 3 has been associated with serious outcomes and complications [8]. PIV can also be clinically significant in ICU and PICU patients [9, 10]. However, not much is known about the burden of PIV infections among ICU and PICU patients. Such data are essential because they could shed light on the importance of these infections and could help researchers and public health officials determine the need for new vaccines and effective antiviral drugs. Until now, no specific antiviral drugs or effective vaccine are available despite the progress made in these fields recently [11–13]. The aim of this study was to evaluate the possible effect of PIV infections on ICU and PICU patients in Kuwait, in addition to defining the clinical features of PIV infections among these patients over a 3-year period.

## Methods

Patient records from the Virology Unit (VU), Faculty of Medicine, Kuwait University Database, were retrospectively reviewed to identify patients with viral infections admitted to the PICU & ICU from January 2013 to December 2015. The VU is an academic institution serving five hospitals in Kuwait (Amiri Hospital, Mubarak Al-Kabir Hospital, Sabah Hospital, Farwaniya Hospital, and Adan Hospital).

### Database and study population

Only Patients with viral infections were included in this study. Data extracted from the records included demographic characteristics, clinical diagnosis, immune deficiency, and number and types of viruses isolated. As this study was retrospective, it did not require ethical approval. These patients were investigated for bacterial, fungal and parasitic pathogens to rule out infection and co-infection. No bacterial, fungal and parasitic pathogens were detected in these patients.

All PIV-infected patients were immunocompetent which was defined as the absence of organ transplantation, human immunodeficiency virus (HIV) or acquired immunodeficiency syndrome (AIDS), autoimmune disease, leukemia, pancytopenia, lymphoma or under immunosuppressive drug therapy. All patients underwent a respiratory virus panel screening. Respiratory system samples (bronchoalveolar lavage or tracheal aspirate) were collected in disposable mucus extractors (Vygon SA, Écouen, France). Samples were assayed by reverse transcriptase-polymerase chain reaction (RT-PCR) to detect 20 respiratory pathogen which include: influenza virus A (Flu A), influenza virus A H1N1 (H1N1), influenza virus B (Flu B), human coronavirus (HCoV)-NL63, -229E, -OC43 and HKU1, PIV-1, -2, -3, -4 human metapneumovirus (hMPV)-A and -B, rhinovirus (HRV), respiratory syncytial virus (RSV)-A and B, adenovirus (AdV), enterovirus (EV), parechovirus (HPeV), human bocavirus (HBoV), using FTD^®^ Respiratory Pathogens kit (Fast-Track Diagnostics Ltd., Sliema, Malta).

### Inclusion and exclusion criteria

Viral studies were not taken routinely and were undertaken only on a clinical basis in patients with suspected viral infections. The patients were divided into three groups: 1) individuals with single documented viral infection during their hospitalization (single); 2) those that were diagnosed with double viral infections during their hospitalization (double); and, 3) those that were diagnosed with triple or more viral infections during their hospitalization (triple). The double and triple infections were detected simultaneously from the same clinical sample provided.

### Statistical analysis

Data analysis was performed using the Statistical Package for the Social Sciences software (SPSS v20.0; IBM Corp, Armonk, N.Y. USA). Descriptive statistics for continuous variables were compared using the nonparametric Mann-Whitney U test or the Kruskal-Wallis test. For categorical variables, the χ2 test, the Fisher exact test or the Z test was applied to evaluate the difference between proportions or to assess whether there were any associations between the proportions. A two-tailed probability value *p* < 0.05 was considered statistically significant.

## Results

Table 1 shows the demographic characteristics and clinical parameters of 1510 patients admitted to ICU (37.5%) and PICU (62.5%) with viral infections. A total of 39 (2.6%) patients were found to have confirmed PIV infections, with 21 (1.4%) in ICU and 18 (1.2%) in PICU. The most frequently isolated PIV type was type 3 (28, 71.8%) followed by type 1 (9, 23.1%). The least frequently isolated PIV type was type 4 (2, 5.1%), and type 2 was not detected. Single infections were identified in 26 patients (66.6%), double co-infection in 9 patients (23.1%) and triple co-infection in 4 patients (10.3%). For double co-infection category, the most frequently isolated virus with PIV was HRV (*n* = 6; 15.4%) followed by Flu A, AdV, and RSV (*n* = 1; 2.3% each). For triple co-infection category, Flu A and HRV were identified in all four patients.

**Table 1:** Characteristics of the study population

	ICU Patients No. (%)	PICU Patients No. (%)	Total
Overall Patients Tested	566 (37.5)^a^	944 (62.5)^a^	1510
PIV-positive	21 (53.9)^a^	18 (46.1)^a^	39 (2.6)^a^
Sex			
Male	17 (43.5)	4 (10.3)	21 (53.8)^b^
Female	7 (17.9)	11 (28.2)	18 (46.2)^b^
Isolation			
PIV-1	7 (18)	2 (5.1)	9 (23.1)^b^
PIV-2	0	0	0
PIV-3	14 (35.9)	14 (35.9)	28 (71.8)^b^
PIV-4	2 (5.1)	0	2 (5.1)^b^
Mixed infection			
Single	14 (35.9)	12 (30.8)	26 (66.6)^b^
Double	6 (15.4)	3 (7.7)	9 (23.1)^b^
HRV	4 (10.3)	2 (5.1)	6 (15.4)^b^
FluA	1 (2.3)	0	1 (2.3)^b^
AdV	1 (2.3)	0	1 (2.3)^b^
RSV	0	1 (2.3)	1 (2.3)^b^
Triple	2 (5.1)	2 (5.1)	4 (10.3)^b^
HRV	2 (5.1)	2 (5.1)	4 (10.3)^b^
FluA	2 (5.1)	2 (5.1)	4 (10.3)^b^

^a^The number in parentheses represents % of patients tested in relation to the total number of patients tested (*n* = 1510)

^b^The number in parentheses represents the number of PIV infections in relation to the total number of PIV infections (*n* = 39)

The age of the patients ranged from 4 days to 71 years with a mean of 14.6 ± 23.4 years and Median (Inter-Quartile) is 7 months (2 months–24.5 years) (Table 2). Table 1 shows age grouping of the study population according to sex. The male to female ratio was 1.2:1.

**Table 2:** Age groups of study population according to gender

Age (years)	Mean ± SD	Median (Inter-Quartile)	Range
Male	19.0 ± 25.9	1 year (52 days–41.5 years)	4 days–71 years
Female	7.2 ± 16.9	4 months (55 days–6.5 years)	21 days–70 years
Overall	14.6 ± 23.4	7 months (2 months–24.5 years)	4 days–71 years

PIV infections were identified in 20 (51.3%) of infants below 1 year of age, 4 (10.3%) below 5 years of age, 7 (18%) patients below 14 years of age, 7 (18%) patients below 59 years of age and 5 (12.8%) ≥59 years of age (Table 3). The most affected age group was infants’ ≤5 months of age 18 (46.2%).

**Table 3:** Age groups according to PIV types in 39 patients

Age group	PIV-1 No. (%)	PIV-2 No. (%)	PIV-3 No. (%)	PIV-4 No. (%)	Total No. (%)
≤1 M*	0	0	7 (7.7)	1 (2.6)	8 (20.5)
2–5 M	2 (5.1)	0	8 (26.5)	0	10 (25.6)
6–11 M	0	0	2 (5.1)	0	2 (5.1)
1–4 Y**	1 (2.6)	0	2 (5.1)	1 (2.6)	4 (10.3)
5–14 Y	1 (2.6)	0	2 (5.1)	0	3 (7.7)
15–29 Y	0	0	1 (2.6)	0	1 (2.6)
30–44 Y	3 (7.7)	0	1 (2.6)	0	4 (10.3)
45–59 Y	1 (2.6)	0	1 (2.6)	0	2 (5.1)
≥60 Y	1 (2.6)	0	4 (10.3)	0	5 (12.8)
All ages	9 (23.1)	0	28 (71.8)	2 (5.1)	39

*M=Month(s)

**Y=Year(s)

Table 4 shows frequency of PIV types in the 39 ICU and PICU patients in relation to symptoms. The majority of the infections caused by the PIV types affected the lower respiratory tract (35 patients, 89.7%) than the upper respiratory tract (2 patients, 5.1%). Pneumonia was the most frequent reason for hospitalization (12 patients, 30.8%), *P* < 0.05, followed by bronchiolitis (10 patients, 25.6%) and chronic obstructive pulmonary disease (COPD) (7 patients, 18%).

**Table 4:** Frequency of PIV types in 39 ICU and PICU patients in relation to symptom presentation

	ICU Patients No. (%)				PICU Patients No. (%)				Total
	PIV-1	PIV-3	PIV-4	Total	PIV-1	PIV-3	PIV-4	Total	
Pneumonia	4 (10.3)	0	0	4 (10.3)	0	8 (20.5)	0	8 (20.5)	12 (30.8)^*^
Bronchiolitis	0	4 (10.3)	0	4 (10.3)	2 (5.1)	4 (10.3)	0	6 (15.4)	10 (25.6)
^a^COPD	3 (7.7)	4 (10.3)	0	7 (18)	0	0	0	0	7 (18)
Bronchitis	0	(10.3)	2 (5.1)	6 (15.4)	0	0	0	0	6 (15.4)
Laryngitis	0	0	0	0	0	2 (5.1)	0	2 (5.1)	2 (5.1)
Fever, Sepsis	0	0	0	0	0	2 (5.1)	0	2 (5.1)	2 (5.1)

^a^COPD: Chronic obstructive pulmonary disease

^*^ *P* < 0.05

**Fig. 1:**
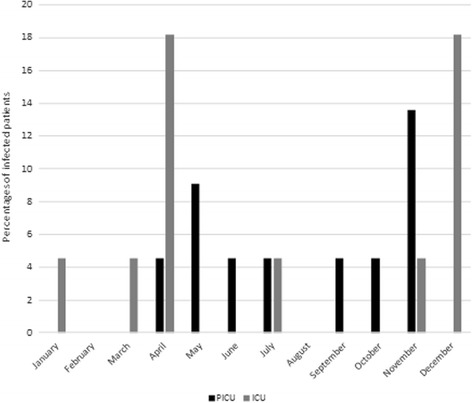
Monthly distribution of PIV infections in ICU and PICU

The monthly distribution of PIV infections was highest during the period from April to May and November to December. The lowest levels were detected in February and August (Fig. 1). The incidence of PIV infections in ICU patients was highest during the months of April and December and PICU patients during the month of May and November. Infection in ICU patients peaked in April (18.2%) and PICU patients in November (13.6%).

**Fig. 2:**
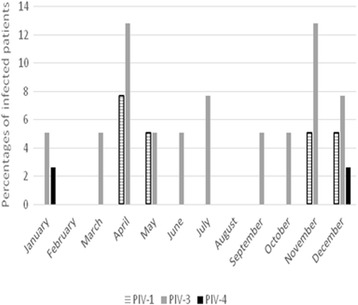
Monthly distribution of PIV-1, PIV-3, and PIV-4 infections

The monthly distribution of PIV infections according to the types isolated is shown in Fig. 2. The incidence of PIV-1 peaked in April (7 patients, 18%), followed by May, November, and December (5 patients, 12.8% each). PIV-3 was present throughout the year except in February and August. The incidence of PIV-3 peaked in April and November (13 patients, 33.3% each), followed by July and December (7 patients, 18% each).

## Discussion

This is a population-based retrospective study aimed at characterizing the impact of PIV infections among infants, children and adults admitted to the PICU and ICU in 5 centers across Kuwait over a 3-year period. This study was performed within five centers to give it a potential strength, which emphasizes on the generalizability of the results for the whole country. Our data highlighted the importance of PIV as a significant cause of respiratory tract infections and disease in ICU and PICU patients which should not be overlooked by both physicians and public health officials. As we found in Kuwait, PIV infections were identified among 2.6% of all ICU and PICU admissions, 1.4% among ICU and 1.2% among PICU patients. Multiple studies from across the world have found that PIV is associated with hospitalization of patients with respiratory tract infections, such as those from Kuwait [14], Saudi Arabia [15]; Jordon [16]; United States [17, 18], China [19, 20], Thailand [21], Iran [22], Bangladesh [23], Kenya [24], South Korea [9], Mexico [25] and Brazil [26]. Although, PIV are considered as a significant causative agents of both upper respiratory tract infections (URTI) and lower respiratory tract infections (LRTI) in infants and young children [3, 12, 27], the importance of these viruses has been underestimated [28].

Previous studies have described different incidences of PIV infections, but these studies involved different study populations and used different detection techniques. A study from Saudi Arabia, investigated the incidence of PIV in hospitalized infants, children aged up to 10 years only during the winter season by direct immunofluorescence assay documented PIV infections in 15.6% of hospitalized patients [15]. In another study by Peltola et al. [29], exploring the etiology of croup in hospitalized children, documented PIV-1, in 29.1% of patients. A study from Spain, involving hospitalized children aged up to 14 years with acute respiratory tract infections, PIV infections accounting for 11.8% of the positive cases [30]. In a study from Brazil aiming to define the etiology of community-acquired pneumonia in hospitalized children younger than 5 years, PIV infections were detected in 3.81%, ranked third among the most dominant viral agents [31].

As documented by Oh [32], we found that children under 5 years of age had greater hospitalization rates, possibly because their respiratory and immune systems are immature, predisposing to severe respiratory tract infections/disease [32]. In this study, the chance of isolating PIV-1, PIV-3, or PIV-4 in PICU patients aged 5 months or younger (46.2%) was three times higher than that in children between 6 months to 4 years of age (15.4%). However, our study showed that 28.2% of PIV-infected ICU patients were older than 30 years.

Mixed viral infections were detected in our study with 33.3% (13/39) of positive cases with co-infections. This rate is higher than those previously described in which co-infection rates ranged from 3 to 20% [31, 33–37]. Earlier studies have described the different incidence of mixed infections probably due to dissimilar study populations and detection techniques. Additional research is essential to address high rates of mixed-infections with other respiratory viruses and to decide if the mixed-infections could result in enhanced severity of PIV infections. In young children, PIV infections are the most common cause of croup, bronchiolitis and pneumonia [6, 38, 39]. In ICU patients, COPD and bronchitis seem to be the most prominent symptoms. PIV, are frequently detected in COPD and bronchitis in the adult population [40–43].

There are four serotypes each with different genetic and antigenic properties, and they vary in clinical picture, incidence, and seasonality [44, 45]. We showed the presence of the three types of PIV infections in Kuwait among ICU and PICU patients, including PIV-4, which was identified for the first time during 2013–2015. PIV-4 has been associated with mild infections [46, 47]. Other studies have shown that it can cause severe illnesses in some settings [48–50]. Though, none of these studies from our region describe the presence of PIV-4 [15, 16, 22]. The incidence of PIV-3 was higher than those of PIV-1 and PIV-4 in this study, as found in other studies [30, 51–53], whereas PIV-2 was not detected. It is documented that PIV-3 is the type most commonly identified in hospitalized patients with pneumonia and bronchiolitis [3, 54].

Outbreaks of respiratory virus infections in countries with meditation or desert climates were documented during the cold season, whereas in tropical countries, they seem to be more associated with the rainy period [55]. The seasonal occurrences of PIV types differ from place to place, an outcome attributed to climatic changes [31]. In a study from Saudi Arabia, Only PIV-3 infection was detected all year round, but epidemics occurred during summer (June–August) [56]. In the USA, biannual patterns were described for PIV-1 and annual peaks for PIV-3 [53, 57]. The seasonal incidence of PIV-1 and PIV-3 among critically ill patients in Kuwait occurred twice-a-year during autumn (April to May) and winter (November to December), and once-a-year during winter (January to December) for PIV-4 (Fig. 2).

## Conclusion

This study highlighted the importance of PIV as a causative agent of respiratory tract infection in critically ill patients, mainly in developing countries, where few data concerning respiratory viruses are available. Further studies may better define the burden of PIV infections and the need for effective measures for prevention and treatment strategies.

## Abbreviations

AdV: Adenovirus
AIDS: Acquired immunodeficiency syndrome
COPD: Chronic obstructive pulmonary disease
Flu A: Influenza A virus
HIV: Human immunodeficiency virus
HRV: Rhinovirus
ICU: Incentive Care Unit
LRTI: Lower respiratory tract infections
PICU: Pediatric intensive care unit
PIV: Parainfluenza viruses
RSV: Respiratory syncytial virus
RT-PCR: Reverse transcriptase-polymerase chain reaction
URTI: Upper respiratory tract infections
VU: Virology unit
